# Diagnosis and management of chronic cough: similarities and differences between children and adults

**DOI:** 10.12688/f1000research.25468.1

**Published:** 2020-07-22

**Authors:** Miles Weinberger, Manju Hurvitz

**Affiliations:** 1 Rady Children’s Hospital, University of California San Diego, San Diego, CA, USA

**Keywords:** Chronic cough, habit cough, tracheomalacia, bronchoscopy

## Abstract

Cough is a natural process that protects the airway. Cough can occur spontaneously or voluntarily. It is considered chronic when it is present for longer than 4 weeks in children or 8 weeks in adults. In both, chronic cough causes patient distress and increased healthcare utilization. Etiologies of pediatric chronic cough include asthma, protracted bacterial bronchitis, tracheomalacia, habit cough, and various systemic disorders. While some diagnoses are identifiable by careful history alone, others require testing guided by specific pointers. Flexible fiberoptic bronchoscopy has been an important tool to identify etiologies of chronic cough that were not otherwise apparent. In adults, asthma and bronchitis are well-defined etiologies of chronic cough, but much chronic cough in adults is largely a conundrum.

Reviews of adult chronic cough report that at least 40% of adults with chronic cough have no medical explanation. Gastroesophageal reflux and upper airway cough syndrome (a.k.a. post-nasal drip) have been common diagnoses of chronic cough, but those diagnoses have no support from controlled clinical trials and have been subjected to multiple published critiques. Cough hypersensitivity is considered to be an explanation for chronic cough in adults who have no other confirmed diagnosis. Gabapentin, a neuromodulator, has been associated with a modest effect in adults, as has speech pathology. While habit cough has not generally been a diagnosis in adults, there is evidence for a behavioral component in adults with chronic cough. Treatment for a specific diagnosis provides a better outcome than trials of cough suppression in the absence of a specific diagnosis. More data are needed for chronic cough in adults to examine the hypothesized cough hypersensitivity and behavioral management. This article reviews etiologies and the treatment of chronic cough in children and the conundrum of diagnosing and treating chronic cough in adults.

## Introduction

Cough is a natural process that can protect the airway. Stimulation of the pharyngeal area will induce cough to prevent a foreign substance from entering the airway. Irritation of cough receptors in the airways can be stimulated by infection, presence of mucus, or foreign material. Cough is characterized as being “wet” based on the presence of mucus heard or produced. Retention of mucus or the sound of mucus in the airway causes the wet cough, while a non-productive cough is considered dry. The sound of cough comes from the sudden release of air compressed by a previously closed glottis. The sequence is inspiration, glottic closure so that pressure is built, followed by the rapid release of the air. The sound of the cough can vary considerably in pitch, timbre, or loudness. Cough can have a loud barking quality or be so soft that it is essentially a throat-clearing sound. When cough continues daily for more than 4 weeks in children, it is considered to be chronic ^1^. The convention in the literature for adults is 8 weeks to define a cough as chronic ^2^.

## Diagnosing the cause of chronic cough in children

A prudent approach to identifying the cause of a chronic cough is beginning with the simplest and least-invasive diagnostic measure ( Table 1). Progression to more complex and invasive measures should be considered if the diagnosis is not apparent from clinical observation and a careful history. It may be tempting to first consider the most common cause of chronic cough in children or the causes of chronic cough with which the clinician is most familiar. While that may seem logical, an incorrect assumption of the etiology and treatment decision results in the frustration of uninformative testing and failed therapeutic trials. A preliminary physical examination and spirometry for those able to perform the procedure can identify auscultatory and physiological abnormalities that may be related to the chronic cough and provide some direction for further evaluation. However, the etiology of chronic cough in children can generally be identified most accurately and efficiently by beginning with observation, a careful history, and progressing to appropriate tests and therapeutic trials based on pointers obtained in the history, radiology, bronchoscopy, and bronchoalveolar lavage when the diagnosis has not been made from those initial measures ^3^.

**Table 1.** A logical approach to the diagnosis of chronic cough for children. The simplest and least-invasive diagnostic tool is history, which can make the diagnosis of habit cough (A) and provides historical pointers to consider diagnoses that require specific testing or therapeutic trial (B–F).

A. Observation and history: frequency of cough—repetitive cough absent once asleep indicates likelihood of habit cough syndrome B. Cough present for <3 months, especially if spasmodic and disturbs sleep, requires consideration of pertussis syndrome C. Cough in infant with feeding warrants textured swallow study D. Cough present since neonatal period, history of transient tachypnea of newborn, and chronic otitis media warrants consideration of primary ciliary dyskinesia E. Cessation of cough after a short course of an oral corticosteroid is consistent with asthma; further evaluation can determine an appropriate treatment plan. Failure to stop cough with the oral corticosteroid warrants further evaluation F. A 2-week therapeutic trial of amoxicillin-clavulanate can be considered as an alternative to bronchoscopy and lavage if the historical pointer suggests a “wet” cough in an infant or toddler in making a clinical diagnosis of protracted bacterial bronchitis 1. Radiology, chest X-ray or computerized axial tomography a. Lobar hyperinflation suggests retained foreign body—consider rigid bronchoscopy b. Suggestion of airway inflammation or bronchiectasis warrants sweat chloride testing for cystic fibrosis c. Situs inversus totalis suggests a likelihood of primary ciliary dyskinesia d. Normal chest X-ray warrants further evaluation 2. Flexible fiberoptic bronchoscopy with bronchoalveolar lavage can determine the following: a. Airway malacia, trachea or bronchi b. Protracted bacterial bronchitis

A diagnosis that can be made by history and observation alone is the habit cough ^4^. This disorder has been repeatedly described in children since 1966 ^5^, sometimes with different terminology, including tic ^6^, tic-cough ^7^, psychogenic cough ^8, 9^, and somatic cough disorder ^10^. It is seen at major pediatric centers about 7–9 times per year ^7, 11^. Regardless of the term used, the same description occurs in repeated publications of a characteristic sound of the cough, frequently barking or honking, and the absence of the cough once asleep ^5, 8, 9, 11– 14^. This is most common in children aged 8 to 13 years (mean and median, 10 years) ^11^ but has been seen as young as 3 and as old as 16. There is a male predominance, with 58% of males among 140 diagnoses of habit cough over a 20-year time span at the University of Iowa and 69% of 55 children over a 6-year period at the Brompton Hospital in London ^7, 11^. Classically described as a barking cough, different sounds have been heard in these patients, including a soft cough consistent with throat clearing in about 10% ^11^. Frequent repetitive coughing is characteristic and can occur multiple times per minute, even with every exhalation in some. Others may have spasms of multiple coughs every few minutes. The repetitive cough may be present for hours on end and even during all waking hours. While interference in getting to sleep from this cough is common, a *sine qua non* for the diagnosis of the habit cough is the absence of cough once asleep ^14^.

Another diagnosis that is important to consider by history and observation is pertussis (whooping cough) ^15^. The cough is characterized by frequent spasms of coughing followed by nausea or vomiting. The cough can persist for up to 3 months. The frequency and sound of the coughs may be like the barking cough often present with habit cough, but the usual presence of troublesome nocturnal coughing distinguishes this chronic cough from habit cough.

Other aspects of history can point to diagnoses that require specific testing. Chronic wet or productive cough can include asthma ^16^, protracted bacterial bronchitis ^17^, cystic fibrosis ^18^, primary ciliary dyskinesia ^19^, or bronchiectasis ^20^. Symptoms of asthma and cystic fibrosis can be apparent at any age. Protracted bacterial bronchitis is most common in the infant and toddler ^21^. Cough in primary ciliary dyskinesia typically begins in infancy and persists ^19^. Bronchiectasis most commonly occurs from the damage caused by the other chronic lung diseases but also occurs in the absence of another lung disease ^20^. The etiologies of chronic cough in children referred to a specialty center have been identified using a standardized management pathway ^21^.

Cough in asthma is commonly associated with recurrent wheezing and dyspnea, but asthma can be manifested in some cases only with cough and is then called cough-variant asthma or cough-dominant asthma ^22^. Asthma is readily identified in both children and adults by a diagnostic therapeutic trial of a short course of prednisone or other systemic corticosteroid ^22, 23^. Pavard and Chung specifically stated “guidelines recommend a carefully controlled 2-week trial of oral prednisone” ^24^. Similarly, Morice *et al*. indicated that a therapeutic trial of prednisolone should be offered if diagnoses of cough-predominant asthma or eosinophilic bronchitis are being considered ^25^. Cessation of cough from the corticosteroid supports the diagnosis of asthma, while failure to stop the chronic cough eliminates the diagnosis and justifies further evaluation. An alternative recommendation from the Global Initiative for Asthma (GINA) was for a diagnostic trial of a short-acting bronchodilator (SABA) and low-dose inhaled corticosteroid for 2–3 months ^26^. Others have recommended just an inhaled corticosteroid for a shorter period to assess if cough is from asthma or using a bronchoprovocation test to examine for the presence of airway reactivity. The GINA recommendation is more expensive, is more problematic for young children, and provides less assurance of successful medication delivery, which is essential for making a diagnostic decision. Examining for airway reactivity is also not an efficient or reliable means of asthma diagnosis ^27^.

Cystic fibrosis has a wet cough. Most common in people of Northern European descent, cystic fibrosis occurs in about 1 in 3,000 of the U.S. Caucasian population. Cystic fibrosis is generally diagnosed in childhood and primarily at the newborn stage because of universal newborn screening, but diagnosis is sometimes delayed until adulthood ^28^. Diagnosis is by measurement of sweat chloride concentration and genetic identification of deleterious mutations of the gene controlling the cystic fibrosis transmembrane regulator (CFTR), a protein that affects functioning in the sweat glands, respiratory system, digestive system, and reproductive system ^29^.

Primary ciliary dyskinesia can be suspected from a chronic wet cough at any age that begins in infancy and persists ^19^. Its prevalence in the U.S. is estimated to be at least 1 in 10,000, much less common than the prevalence of cystic fibrosis. A history of transient neonatal distress is common, thought to be a result of decreased clearance of amniotic fluid from the lungs because of the absence of normal ciliary function. The absence of ciliary action *in utero* causes random assignment of the organs ^30^. Consequently, about 50% of those with primary ciliary dyskinesia have situs inversus totalis. The disorder has been known as Kartagener’s syndrome ^31^.

A screening test for primary ciliary dyskinesis is measuring nitric oxide from the nose ^32^. Primary ciliary dyskinesis is characterized by extremely low values of nasal nitric oxide. A normal to high value of nasal nitric oxide excludes the diagnosis. Confirmation of the diagnosis can be made by demonstration of defects in the dynein arms of the cilia when seen by electron microscopy or identification of defects in genes that control components of the cilia ^33^. But, currently, both identify only about 70% of cases. Examining ciliary movement by high-speed video-microscopy analysis is used to aid the diagnosis of primary ciliary dyskinesia. This technique enables the identification of absent or dysfunctional ciliary movement.

Radiology can point to various diagnoses. Suggestion of bronchiectasis on plain films can be confirmed with chest computerized tomography (CT). Bronchiectasis occurs with several chronic lung diseases, such as cystic fibrosis and primary ciliary dyskinesia, and early signs of bronchiectasis have been reported in some patients with protracted bacterial bronchitis ^34^. Bronchiectasis unrelated to chronic lung disease is also seen ^20^.

Flexible bronchoscopy and bronchoalveolar lavage are essential to confirm a diagnosis of protracted bacterial bronchitis, a clinical disorder predominant in infants and toddlers. This disorder has been the most common cause of chronic cough referred to specialty centers. Neutrophilia and greater than 10,000 bacteria/mL in bronchoalveolar fluid are the diagnostic findings ^17^. The organisms involved are *Hemophilus influenzae*, *Moraxella catarrhalis*, and *Streptococcus pneumoniae*, the same organisms involved in otitis media. Clinical response to a 2-week course of amoxicillin-clavulanate is also used as a presumptive diagnostic criterion when the clinical presentation is consistent with protracted bacterial bronchitis. Diagnosis of PBB made by the contents of a bronchoalveolar lavage are then identified as PBB-micro, whereas a diagnosis made based on a clinical response to antibiotics is identified as PBB-clinical ^35^. Flexible bronchoscopy, when done properly, entering through the nose with controlled sedation ^36^, can identify tracheobronchomalacia ^37^ and some uncommon causes of chronic cough ^38, 39^.

Tracheomalacia or tracheobronchomalacia is a disorder that can occasionally cause chronic cough ^40^. It involves tracheal or main stem bronchus collapse to a variable degree. It can be diagnosed only by bronchoscopy performed with light sedation so that dynamic movements can be visualized. Most tracheomalacia or tracheobronchomalacia cause minimal problems. When the collapse of the trachea results in the anterior and posterior walls of the trachea coming into contact, this can result in a nidus of irritation that causes chronic cough ^41^.

Other causes of chronic cough in children include foreign body aspiration ^42^, which may cause localizing auscultatory findings. An uncommon cause of chronic cough in children is of otogenic etiology ^43^, when a foreign body in the ear stimulates Arnold’s nerve. Various chronic lung diseases can also be associated with chronic cough but will usually manifest other findings such as tachypnea and hypoxemia that lead to specific investigations.

## Treatment of chronic cough in children

Treatment is dependent on the correct diagnosis. Children with habit cough are often inappropriately treated because the diagnosis is not recognized and treatment is given for other causes of cough with which the physician is more familiar ^4^. However, the diagnosis should not be one of exclusion by eliminating other causes of chronic cough first. The clinical presentation of habit cough, a repetitive cough absent once asleep, is sufficiently characteristic to make the diagnosis without further testing or therapeutic trials ^7, 11^. Recognizing the diagnosis then permits treatment using some form of suggestion therapy, which is generally curative ^12– 14^. Cough from asthma is readily relieved by a short course of an oral corticosteroid ^22^. Cessation of cough by an oral corticosteroid justifies further evaluation to characterize the asthma and consider maintenance medication, such as an inhaled corticosteroid.

Pertussis warrants treatment with a macrolide antibiotic, preferably azithromycin. This eliminates the serious contagiousness of this disorder. However, the clinical course is little affected beyond early treatment ^44^. Cystic fibrosis is a multi-system disease. Treatment requires pancreatic enzyme replacement therapy for most ^45^. Antibiotics, mucolytic agents, antibiotics, and airway clearance therapy are used to minimize the progressive airway damage that occurs from chronic infection ^18^. New medications identified as potentiators and correctors can restore CFTR function ^46^.

Treatment of bronchiectasis focuses on the primary disease such as cystic fibrosis or primary ciliary dyskinesia. Mild cylindrical bronchiectasis may be reversible with treatment ^20^. It can persist as a disease-causing chronic purulent cough even if the defect causing the disease is pharmacologically restored and progression of the disease is limited. This is now occurring with cystic fibrosis where the bronchiectasis, if previously present, remains even after the CFTR function is restored ^46^ Treatment of bronchiectasis, whether from a primary disease or idiopathic, involves airway clearance therapy and antibiotics.

Treatment of primary ciliary dyskinesia has some similarities to that of cystic fibrosis, but the mucus is not as thick and sticky as in cystic fibrosis and responds better to drainage by airway clearance therapy ^19^. Antibiotics become more important as the disease progresses with the development of bronchiectasis, though at a slower rate than cystic fibrosis.

Protracted bacterial bronchitis generally responds well to 2 weeks of amoxicillin-clavulanate ^17^. Sometimes a longer or repeat course is needed, and frequent recurrences occur requiring repeated treatment. Tonsillectomy eliminates cough from tonsils impinging on the epiglottis ^39^. A rare cause of a uvula in contact with the epiglottis causing cough was cured by uvulectomy ^38^. The cough of tracheomalacia has a barking quality, much as frequently occurs in habit cough, but persists during sleep, unlike habit cough ^37^. When the cough from tracheomalacia is sufficiently severe and intractable, a procedure known as aortopexy is considered ^47^.

Thus, using a systematic approach, the etiology of chronic cough in children is generally diagnosable. Treatment is dictated by the diagnosis. Chronic cough without explanation is rare in children. For the few not initially diagnosable, new etiologies continue to be found ^48^.

## Diagnosing the cause of chronic cough in adults

In contrast to the identification of chronic cough in children, chronic cough in adults often remains a conundrum. Asthma is an occasional isolated cause of chronic cough in the absence of other symptoms of asthma for both children and adults ^49^. Since asthma, including cough-variant asthma, is a common cause of chronic cough, a course of oral prednisone can be given as a therapeutic trial for both adults and children ^22– 25^. The response provides diagnostic information. Failure to respond indicates the absence of asthma. Response supports the diagnosis of asthma and warrants consideration for further evaluation and treatment for asthma. Spirometry can provide evidence to support the diagnosis of asthma by demonstrating bronchodilator responsive airway obstruction. Other etiologies commonly attributed to chronic cough in adults, gastroesophageal reflux and upper airway cough syndrome (also known as post-nasal drip), are not seen as a cause of chronic cough in children submitted to a standardized assessment ^21^. While gastroesophageal reflux and post-nasal drip (or drainage) are extremely common disorders in otherwise healthy adults ^50^, most with these common disorders are not troubled by chronic cough. The incredulousness of these diagnoses as a cause of chronic cough has been repeatedly expressed for children ^51, 52^ and adults ^53– 56^. Vocal cord dysfunction (VCD) is described as causing chronic cough ^2^, but cough was not seen in 49 sequential adolescents with VCD ^57^.

In addition to asthma, angiotensin-converting enzyme inhibitor-induced cough, non-asthma eosinophilic bronchitis, chronic bronchitis, chronic obstructive pulmonary disease, bronchiectasis, pulmonary fibrosis, lung cancer, and obstructive sleep apnea syndrome have been reported to be associated with chronic cough in adults ^58^. A review by the European Respiratory Society suggested that, while a wide range of diseases may be associated with chronic cough in adults, the heightened sensitivity of neuronal pathways provides a common pathway that mediates cough ^59^. Reviews of chronic cough in adults have reported that 40% of chronic cough in adults seen at cough centers is without medical explanation ^2, 60^. Since chronic cough without medical explanation may be termed idiopathic or refractory, cough hypersensitivity as a unifying hypothesis has been an attractive area of investigation ^61^.

An additional specific cause of chronic cough reported for adults is enlarged tonsils ^62, 63^, an observation also made for children ^39^. Tracheomalacia, a cause of chronic cough in some children, can also cause chronic cough in adults. However, the etiologies, surgical options, and outcomes are quite different than in children ^64^. Aspiration of a foreign object, more common in children, has also been a reported cause of chronic cough in adults ^65^. In a Chinese study of adult patients with foreign body aspiration, chronic cough was the most common symptom ^66^. A foreign body in the ear stimulating Arnold’s nerve causing chronic cough has been described in both children ^43^ and adults ^67^. Cough can be present in adults with tuberculosis but is usually not present in primary tuberculosis in children. Chronic cough in adults is common in patients with lung infections due to *Mycobacterium avium* complex, other nontuberculous mycobacteria, fungal diseases, and paragonimiasis ^68^.

## Treatment of chronic cough in adults

The recommended etiologies to be treated in reviews are gastroesophageal reflex and upper airway cough syndrome ^2^. However, there are only anecdotal guidelines and opinions with no controlled clinical trials to support successful treatment of those postulated disorders ^52– 54^. When considering lung diseases such as non-asthmatic eosinophilic bronchitis, chronic bronchitis, chronic obstructive pulmonary disease, bronchiectasis, pulmonary fibrosis, lung cancer, and obstructive sleep apnea syndrome, the use of pointers from history, spirometry, radiology, bronchoscopy, and biopsies may be needed. Tonsillectomy, effective in children where the tonsils impinge upon the epiglottis ^39^, is reported to be an effective treatment for chronic cough in some adults with enlarged tonsils ^62, 63^. Similar to the pediatric report ^38^, an adult with chronic cough was successfully treated by uvulectomy ^69^.

Pharmacologic treatments that modulate neuronal function have been considered for chronic intractable cough in adults when there is no medical diagnosis. Based on the hyperexcitability of neuronal pathways mediating cough, modulating neuronal function has been attempted with morphine, amitriptyline, and gabapentin ^70^. The problem of morphine for a chronic cough is self-evident. Amitriptyline apparently has a transient effect on cough suppression, with fewer patients continuing to benefit with time. In a controlled clinical trial, gabapentin decreased mean cough frequency by about 50% from an initial 45/hour ^71^. The rationale for gabapentin as a treatment for chronic cough is based on the postulation that airway nerve endings in the upper airway create the feeling that causes repeated coughing.

Gefapixant is a medication under investigation with the rationale of greater neuromodulator effect ^72^. Its mechanism is related to being a P2X3 receptor antagonist. P2X3 receptors are found on peripheral sensory nerves of the airways. At a dose of gefapixant that significantly reduced cough at about the same magnitude as gabapentin, adverse effects, primarily dysgeusia and ageusia, occurred in over half of the 63 patients in the study. While the concept of a neuromodulator approach to treatment is intriguing, it has not been determined if the neurogenic stimulus for the feeling that causes repeated coughing is primary or from the continuing stimulation of the cough itself, a classic chicken and egg question.

A non-pharmacologic approach to suppressing chronic cough has included speech and physical therapy. Examined in a controlled clinical trial, a decrease in mean cough frequency from 17 to 9 per hour was described (a magnitude of benefit similar to that seen with gabapentin or gefapixant) ^73^. Dr Mandel Sher, director of a cough center in Florida, noted similarities of habit cough in children and cough identified as neurogenic in adults. Both appear to be triggered by the urge to cough from feeling a sensation in their throat. Both usually occur after a pathologic event, usually a viral respiratory infection. Dr Sher further suggested that the 30–40% placebo response in various trials further indicates a behavioral component in adult chronic cough ^74^. Some adults with chronic cough have reported that their chronic cough stopped from watching a video ( https://www.youtube.com/watch?v=jnQUvD8Qdj0) of a 12-year-old girl with a 3-month history of chronic cough who stopped coughing while receiving suggestion therapy ^75^. Comments received in emails from two adult women, aged 58 and 68, who were among those who stopped coughing after watching the video stated, “ *I listened to the video and concentrated. It really works.*” The other stated, “ *It’s amazing mind over matter*.”

The observation that adults can stop coughing when watching a video of suggestion therapy given to a child suggests a habit cough diagnosis for those adults. However, the presence or absence of the cough when sleeping was not obtained in most of those patients, and absence once asleep is a diagnostic criterion for habit cough. Without further clinical information to support habit cough, the response could be considered a placebo effect ^76^. Interestingly, a publication in a book from 1694 described an “ *habitual cough which often continues after the first cough, which was caused by the cold, is gone, from which….the habitual cough often proceed*” ^77^. This 336-year-old description is similar to habit cough in children and intractable chronic cough in adults. Perpetuating after a viral respiratory infection, as apparently described in 1694, chronic cough may be some form of learned or neurogenic stimulation or damage initiated by the initial pathology.

## Discussion

In comparing chronic cough in children and adults, there are striking differences but also similarities. The most apparent difference is the diagnosis of children with chronic cough by a systematic approach leaves few without a medical explanation ^21^. In contrast, the absence of a medical diagnosis other than the postulate of cough hypersensitivity is common for adults with chronic cough. Consequently, treating a specific diagnosis can occur for children with chronic cough. Without a specific diagnosis other than cough hypersensitivity, treatment of adult chronic cough has focused on pharmacological medication. However, the consensus of expert opinion is that currently available drugs are largely ineffective in the treatment of cough hypersensitivity ^61^. On the other hand, limited evidence suggests that behavioral therapy can provide effective treatment options for adult chronic cough without medical explanation, as has been established for children ^11^. That evidence includes the observations of Dr Mandel Sher ^74^ and the response of some adults to watching a video of a 12-year-old girl being successfully treated with suggestion therapy ^75^. Thus, there is limited value of pharmacotherapy for chronic cough in adults and the similarities to habit cough in children suggest further exploration and use of non-medication behavioral responses for chronic unexplained cough in adults.

Further consideration must also be given to the confounding that might occur when a physical cause of sustained cough, asthma in children or adults, chronic bronchitis in adults, and all other lung diseases that can cause cough, may be accompanied by the repetitive nature of the habit cough overlying the organic cough. Most organic causes of chronic cough do not cause the frequent repetitive cough of habit cough. Treating the underlying disorder as usual and the addition of a behavior method may be needed in such cases.

As a final thought for the reader of this review, consideration should be given to the case described by Smith and Woodcock in the *New England Journal of Medicine* a few years ago ^60^. A 63-year-old woman previously in good health had a 1-year history of a chronic dry cough associated with a sensation of “irritation” in the throat. A history and physical exam were uninformative. She was prescribed a bronchodilator, inhaled and nasal glucocorticoids, all without benefit. She had normal spirometry and chest X-ray. The authors of this case then reviewed causes of chronic cough. Among consideration of diagnostic procedures were a 2-month trial of a proton-pump inhibitor, a methacholine challenge, measurement of the fraction of exhaled nitric oxide, high-resolution CT of the thorax, bronchoscopy, and nasendoscopy. If all were negative, treatment options described included slow-release low-dose morphine sulfate, gabapentin, or speech and language therapy. Meanwhile, the authors of this case provided verbal reassurance to the patient that chronic refractory cough may resolve or decrease spontaneously. In other words, they would kick the can down the street after performing tests and trials involving large sums of money. This case study and recommendations from a prestigious journal warrant careful consideration by readers of this review, not because the clinical course and proposed testing and treatment are atypical and unusual but because they aren’t. It fits an all-too-common pattern of the experiences of children and adults with chronic cough. The authors of this case expressed no knowledge of a disorder with potential relevance to their patient that was described over 300 years ago ^77^ and in children since then ^5, 11, 13, 14^. Utilizing updated information since that ancient report of “habitual cough”, we challenge the reader of this review to think about how they would have managed this lady based on current knowledge.

## Summary

There are multiple causes of chronic cough. A systematic approach involves first a careful history. Habit cough, though less common than other causes, generally can be diagnosed by the history alone. The history in children also provides pointers that lead to specific diagnostic tests for asthma, cystic fibrosis, primary ciliary dyskinesia, protracted bacterial bronchitis, and tracheomalacia. Flexible fiberoptic bronchoscopy can identify etiologies for chronic cough that were not otherwise apparent. Treatment of a specific diagnosis provides better outcome than therapeutic trials of cough suppressants. More and better data are needed for chronic cough in adults in order to select the best therapy. The suggestion of a response to behavioral approaches in adults warrants further investigation.
